# Associations Between Informal Caregiving and Physical Functioning: A Longitudinal Analysis of Dutch Older Adults

**DOI:** 10.1093/geront/gnaf108

**Published:** 2025-03-18

**Authors:** Zeinab Sattari, Dorly Deeg, Louise Meijering, Gerd Weitkamp

**Affiliations:** Department of Demography, Faculty of Spatial Sciences, University of Groningen, Groningen, The Netherlands; Department of Epidemiology and Data Science, Amsterdam Public Health Research Institute, Amsterdam UMC, Vrije Universiteit Amsterdam, Amsterdam, The Netherlands; Department of Demography, Faculty of Spatial Sciences, University of Groningen, Groningen, The Netherlands; Department of Cultural Geography, Faculty Board, Faculty of Spatial Sciences, University of Groningen, Groningen, The Netherlands

**Keywords:** Generalized estimating equations, Healthy aging, Informal care, Longitudinal, Physical functioning

## Abstract

**Background and Objectives:**

Despite a growing body of literature on physical functioning and informal caregiving in later life, few studies have explored how physical functioning changes over time in older caregivers versus noncaregivers and the role of different functioning types in understanding these changes. This study investigates the association between informal caregiving and changes in physical functioning over time among older adults in the Northern Netherlands.

**Research Design and Methods:**

We analyzed data from 2 waves of the Lifelines Cohort Study, using a sample of 9,912 older caregivers and noncaregivers. We examined 11 outcome variables: overall physical functioning and 10 physical functioning types (e.g., vigorous and moderate activities; lifting/carrying groceries; walking various distances). We also controlled for health and demographic characteristics. Associations between changes in physical functioning and caregiving were modeled using generalized estimating equations.

**Results:**

Caregiving affects the effect of aging on older adults’ physical functioning, with caregivers experiencing less decline in overall physical functioning, moderate activities, and lifting/carrying groceries compared with noncaregivers. Despite this, caregivers exhibited higher mental and physical impairments at baseline, contradicting aspects of the healthy caregiver hypothesis. Gender differences were significant, with women showing more limitations in physical functioning than men. Additionally, higher household income and educational attainment were associated with better physical functioning, potentially weakening the negative association between caregiving and aging.

**Discussion and Implications:**

This research contributes valuable insights into healthy aging, informal care, and disability in later life, indicating the need for tailored interventions and policies for older caregivers.

## Background and Objectives

Maintaining or limiting decline in physical functioning is critical for to later life wellbeing, and health-related quality of life (Painter et al., 1999; Ziegler & Schwanen, 2011). Physical functioning encompasses motor function, physical fitness, bodily control, and routine physical activities (Verbrugge & Jette, 1994). These measures provide insights into the ability of older adults to carry out everyday types of physical functioning, such as climbing stairs, walking different distances, cycling, lifting and carrying objects, and bathing and dressing.

Physical functioning declines with age, but the rate of decline is influenced by various factors. For instance, higher levels of physical activity, such as doing sports and exercise in midlife, are associated with slower decline in physical functioning in later life (Chen et al., 2015). Likewise, a weaker social support network is a predictor of greater decline in physical functioning among older adults (Cappelli et al., 2020). Maintaining a healthy lifestyle throughout the life course contributes to both physical and mental well-being, including improved physical functioning, which can promote healthy aging (Kuh et al., 2014). However, certain life situations, such as providing care for a chronically ill or disabled family member or friend, can alter this lifestyle and result in a different physical functioning outcome. Informal caregivers, often termed “hidden patients,” are more susceptible to developing various physical and mental impairments while providing care for others. These impairments can impose extra burdens on both caregivers and care-receivers, while also raising healthcare costs for society as a whole (Sambasivam et al., 2019).

### Physical Functioning and Informal Caregiving in Later Life

In the context of an aging population and a policy preference for aging in place, a rising number of older adults are becoming informal caregivers for their loved ones at home (Wiles et al., 2012). A study on surveys across 15 European member states revealed that 13% of adults in Portugal and Spain, and more than 22% of adults in Luxembourg, Belgium, and Denmark give informal care (Tur-Sinai et al., 2020). Informal caregivers, hereafter referred to as caregivers, are individuals who provide unpaid, ongoing assistance to a relative or a friend with a health impairment.

Caregiving is negatively associated with health. Studies have shown that caregivers are more likely to experience poor mental, cognitive, and physical health compared to noncaregivers (Lavela & Ather, 2010). Women and married caregivers, as well as those offering intensive care, are particularly susceptible to the adverse health effects of caregiving (Bom et al., 2019). The mental stress of caregiving can weaken the immune system and lead to health issues, notably a higher level of inflammation related diseases, among older caregivers in particular (Lovell & Wetherell, 2011). Chronic inflammation is associated with reduced physical functioning in later life (Brinkley et al., 2009). Moreover, mental or emotional stress among spousal caregivers is associated with a 63% higher mortality risk compared to noncaregiving controls (Schulz & Beach, 1999). Despite these negative associations, studies reveal that resilience factors against stress, such as personal mastery, self-efficacy, and coping can mitigate the negative physiological associations of stress with caregivers’ health (Harmell et al., 2011). Furthermore, caregivers with a higher level of physical activity often have better mental health (Bucki et al., 2016).

Although research on the positive effects of caregiving tends to focus on the mental health and cognitive functioning of caregivers (Bhattacharyya et al., 2023; van Campen et al., 2013), there is evidence that some older women caregivers with higher-intensity tasks experience less decline in their physical functioning over two years compared with noncaregivers and lower-intensity caregivers (Fredman et al., 2009). This trend is in line with the healthy caregiver hypothesis, which suggests that many older adults who are already in good health become caregivers, and that ongoing physical and cognitive demands of caregiving may help them withstand the emotional stress of intensive care and maintain their physical and cognitive health (Bertrand et al., 2012; Fredman et al., 2008). Studies have yet to explore the nature and extent of associations between caregiving and specific types of physical functioning, such as walking, climbing stairs, or carrying objects. As caregivers age, the associated types of physical functioning decline may differ from that of noncaregivers, leading to specific difficulties when it comes to performing everyday activities.

### Contextual Elements Influencing Physical Functioning and Caregiving in Later Life

Evidence on the changes in caregiver physical functioning varies based on demographic factors (i.e., age and gender) and socioeconomic factors (i.e., household income and education level), as well as the physical and mental health condition of the caregiver. These contextual elements assist health research and policymaking in understanding which caregivers may be more (or less) susceptible to the adverse associations of caregiving with their physical functioning. Due to the limited body of literature examining the context of the relationship between physical functioning and caregiving in later life, we have also incorporated broader contextual elements of caregiving and physical health.

Pinquart and Sörensen conducted several meta-analysis studies (2005, 2006, 2007) investigating the demographic and socioeconomic context of caregivers’ physical health. These studies show that women caregivers exhibited lower levels of physical health compared with men. Additionally, a higher socioeconomic status was positively associated with caregivers’ physical health. Age as a factor showed both negative and positive correlations with changes in physical functioning. While older caregivers may have experienced more age-related health issues than their younger counterparts, they tended to have fewer noncaregiving life stressors, such as demanding jobs or very young children.

Physical and mental impairments are associated with lower physical functioning among older adults (Garber et al., 2010; van der Windt et al., 2008). Impairments may include clinical anxiety and depression, sleep disturbance, chronic physical morbidity, and the need for care and medication. Caregivers are particularly susceptible to developing mental impairments, but there is no strong evidence showing that they will similarly develop more physical impairments when compared with noncaregivers (Barbosa et al., 2020; Roth et al., 2015). This might be attributed to the healthy caregiver hypothesis, which suggests that caregivers are typically healthier than noncaregivers and have to maintain their health to fulfill their caregiving responsibilities.

Despite the growing body of literature on the link between physical functioning and caregiving, few studies have directly focused on older caregivers and the change in their physical functioning over time. Therefore, our study aims to explore the association between informal caregiving in later life and changes in types of physical functioning over time among older adults living in Northern Netherlands. We aim to answer the following questions:

1. How do changes in physical functioning over time among older caregivers compare with those among noncaregivers?
2. Can distinguishing between various types of physical functioning, such as vigorous and moderate activities, shed light on differences in changes over time?

Understanding changes in physical functioning between older caregivers and noncaregivers is crucial for promoting healthy aging and mitigating the costs associated with declining physical functioning for individuals and society. Moreover, providing insight into a combination of physical functioning types can enable research and practice to understand how individuals move around and perform daily activities. Focusing on changes in various types of physical functioning allows research and practice to understand how each type may change at different rates over time, suggesting different interventions to prevent further and rapid decline.

## Research Design and Methods

### Participants and Procedure

This study forms part of the Meaningful Mobility project, which investigates later life mobility patterns and experiences in relation to wellbeing using qualitative and quantitative methods. For this study, we obtained the Lifelines cohort data (Scholtens et al., 2015). The data, collected from more than 167,700 participants, constitute a substantial population-based cohort and biobank created to explore factors influencing the process of healthy aging in the Northern Netherlands. Lifelines consistently produces extensive phenotypic and genotypic data about the general population, facilitating investigations into the progression of chronic and rare diseases, along with their associated sociodemographic, biological, behavioral, and psychological factors. The overarching aim is to make significant contributions to the understanding of healthy aging, public health, and medicine. The collection, management, and use of Lifelines data comply with the ethical principles of the Declaration of Helsinki and with the research code of the University Medical Center Groningen (UMCG). Consequently, the results of our study do not disclose any personal information about the participants.

Initial surveys of physical functioning, caregiving status, health, and demographic characteristics of participants were conducted between 2008 and 2014, during the baseline wave of Lifelines study. Repeat surveys were conducted at wave 2 (W2), between 2019 and 2023. Our study included individuals who were aged 55 years and older at baseline and who provided answers to all the survey questions included in our analysis.

At baseline, 32,201 participants were aged 55 or above. Of these older adults, 19,466 provided complete answers to the physical functioning, caregiving, and covariate questions. At W2, 9,554 participants were excluded because they did not provide complete answers to the physical functioning questions (*n* = 7,845) or they passed away before W2 (*n* = 1,709; 74% noncaregivers). The final sample for our study consisted of 9,912 older adults.

### Caregiving

We identified being a caregiver versus a noncaregiver as the main independent variable of change in physical functioning. Two questions from the Lifelines survey were used to determine whether or not a participant was a caregiver. Respondents who answered one or more to the question “On average how many hours per week do you spend caring for one or more family members?” were coded as caregivers, and those who answered zero were coded as noncaregivers. This approach resulted in a large amount of missing data. To address this issue, participants who answered “yes” or “no” to the question “I do not provide care for family members, friends, or neighbors; yes or no?” were added to the sample. They were then classified as noncaregivers and caregivers, respectively.

### Definition and Types of Physical Functioning

Eleven outcome variables were used: 10 types of physical functioning and a total physical functioning score. Physical functioning was measured using self-reports to 10 questions, which gauge the degree of limitation in various types of physical functioning due to health issues. These questions were derived from the RAND-36 measures of Health-Related Quality of Life (Hays & Morales, 2008). The 10 types of physical functioning consisted of the following: vigorous activities; moderate activities; lifting or carrying groceries; bending, kneeling, or stooping; climbing one flight of stairs; climbing several flights of stairs; walking various distances (100 m, 0.5 km, or more than 1 km); and bathing or dressing (see Supplementary Table 1). The degree of limitation was initially categorized as severely limited, mildly limited, and not limited at all. However, due to two main considerations, this classification was simplified by merging the severe and mild limitations into a single category. First, the original distribution of data exhibited negative skewness. Second, multiple empty categories in an ordinal outcome variable (like the “severe limitation” category in our sample) could potentially reduce statistical power. Consequently, physical functioning types were dichotomized into two categories: limited and unlimited. The total physical functioning score was determined using the guidelines outlined in the RAND-36 survey (Hays et al., 1998). This method involved calculating the average of the various physical functioning aspects, resulting in a continuous variable ranging from 0 to 2. A higher score indicated better overall physical functioning.

To reduce the risk of reverse causality issues, only the outcome data were used longitudinally. Reverse causation arises in cross-sectional studies when the outcome precedes the exposure (Besser et al., 2021). Even though we used observational data in this study and did not aim to draw definitive causal conclusion, we used this strategy to clarify the direction of associations. If we had used all variables longitudinally, it would have been difficult to disentangle whether changes in caregiving status were associated with changes in physical functioning or vice versa.

### Health-Related and Demographic Characteristics

We controlled for the confounding effects of several health-related and demographic characteristics in the association between caregiving and physical functioning. Gender, age, household income, educational attainment, and mental and physical impairment were self-reported at baseline. Age was reported as a continuous variable in years. Educational attainment was scored based on answers to “the highest obtained educational qualification.” Junior secondary education and lower was scored as low educational attainment; secondary vocational to senior general secondary education was scored as medium; and higher vocational education and above was scored as high. Monthly net household income was reported in a large number of categories. These categories were reduced to low (below €1,500), medium (€1,500–€2,500), and high income (over €2,500), based on the distribution of net income in euros at baseline, used by van der Meer (2008).

Mental health impairment was defined as the presence of one or more mental disorders at baseline or in the past; it was coded as not present = 0 and present = 1. These self-reported disorders included depression, anxiety, bipolar, burnout, attention-deficit/hyperactivity disorder, agoraphobia, panic disorder, obsessive-compulsive disorder, and social phobia. The level of physical impairment was determined as a continuous variable based on the number of self-reported physical symptoms of illness (somatic symptoms from the SCL-90 checklist) present at baseline and 7 days prior. This covariate reflects symptoms affecting the autonomic and musculoskeletal systems, linked to an overall sense of physical dysfunction. This method is proven effective for measuring the health condition of older adults (Van Driel et al., 2018). These symptoms consisted of back pain, chest pain, dizziness, dyspnea, headache, heavy limbs, muscle pain, nausea, numbness, feeling alternately hot or cold, and feeling a lump in the throat. The total score for physical impairment ranged from 0 to 12.

### Analysis

The baseline characteristics between caregivers and noncaregivers were compared using Pearson’s chi-square and *t* test statistics. The association between changes in physical functioning and caregiving was modeled using generalized estimating equations (GEE), which is a longitudinal method that accounts for intercorrelation between the two measurement waves. This method is free from regression to the mean, which commonly occurs when using change scores in simple regression models. An exchangeable correlation structure is employed for the GEE model to account for interdependence among repeated measurements within participants. We used linear and binary GEE models to analyze the total physical functioning score and each physical functioning type, respectively. In a stepwise method, regression models were built by adding (a) wave, caregiving, and wave × caregiver; (b) gender and age; (c) household income and educational attainment; and (d) mental and physical health. The “wave” variable indicated the two timepoints. This variable represented the change in physical functioning in noncaregivers (the 0-value of the caregiver variable). The interaction term wave × caregiver indicated the difference between the change in physical functioning of noncaregivers and caregivers between baseline and W2. To obtain the change in caregivers’ physical functioning, the Beta values were added in the case of a linear model, and the odds ratios were multiplied in the case of a binary model.

All models demonstrated a good fit to the data, as evidenced by significant improvements in the chi-square values when compared with the empty model. Additionally, the significant results of the final GEE model were visually represented via graphs, illustrating the change in the estimated marginal mean scores of physical functioning among caregivers versus noncaregivers. A preliminary analysis indicated no risk of multicollinearity between independent variables (all tolerance scores >0.1). All analyses were performed in SPSS.28, with the statistical significance set at *p* < .05.

## Results

Across a total of 9,912 participants, 35% provided care at baseline; 51% were women; the average age was 61.3 years (*SD* = 4.7, range: 55–84); the majority had high (54.2%) or medium (35.1%) household income; 38.4% had low educational attainment, 27.5% had medium attainment, and 34.0% had high attainment; 18% had mental impairment; and the average physical impairment score across all participants was 2.89.

In our study sample, over 65% reported no limitations in all types of physical functioning, except for vigorous activities, which were limited for more than 65% of participants in both waves. The average total score for participants’ physical functioning was 1.78 (*SD* = .28) at baseline and 1.68 (*SD* = .36) at W2.

Table 1 presents the distribution of physical functioning, demographic characteristics, and health status at baseline categorized by caregiving status. Significant differences exist between caregivers and noncaregivers in specific types of physical functioning: there were fewer limitations for caregivers in vigorous activities and more limitations in lifting or carrying groceries, climbing several flights of stairs, and bathing or dressing. Furthermore, a significant gender difference was apparent, with 60.9% of caregivers being women, contrasting with 46.1% of noncaregivers. Additionally, compared with noncaregivers, caregivers tended to be slightly younger on average, achieved medium and high levels of educational attainment, and lived with more mental health impairments.

**Table 1.** Distribution of Physical Functioning, Demographic Characteristics, and Health Status of Participants at Baseline Based on Caregiving Status

Variables		Caregivers (*n *= 3,487)	Noncaregivers (*n *= 6,425)	*p* Value
Outcome variables				
Vigorous activities, such as running, lifting heavy objects, participating in strenuous sports (%)	Limited	65.4	68.2	**.005**
Moderate activities, such as moving a table, pushing a vacuum cleaner, cycling (%)	Limited	15.9	17.3	.071
Lifting or carrying groceries (%)	Limited	19.8	23.9	**< .001**
Climbing several flights of stairs (%)	Limited	23.4	25.3	**.032**
Climbing one flight of stairs (%)	Limited	8.0	7.5	.309
Bending, kneeling, or stooping (%)	Limited	32.8	33.1	.795
Walking more than 1 km (%)	Limited	12.1	12.6	.461
Walking 0.5 km (%)	Limited	6.1	5.5	.230
Walking 100 m (%)	Limited	3.3	2.8	.182
Bathing or dressing yourself (%)	Limited	2.2	1.5	**.023**
Total physical functioning score (mean)	Range 0–2	1.77 (*SD* = .27)	1.78 (*SD* = .28)	.537
Covariates				
Gender (%)	Man	39.1	53.9	**< .001**
	Woman	60.9	46.1	
Age (mean)	Range 55–84	61 (*SD* = 4)	62 (*SD* = 5)	**< .001**
Household income (%)	Low	11.1	10.5	.492
	Medium	35.4	35.0	
	High	53.5	54.5	
Educational attainment (%)	Low	36.2	39.7	**.003**
	Medium	28.7	26.8	
	High	35.1	33.5	
Mental health impairment (%)	No	80.0	81.8	**.013**
	Yes	20.0	18.2	
Physical health impairment score (mean)	Range 0–12	3.02 (*SD* = 2.473)	2.82 (*SD* = 2.442)	.981

*Note*: *n* = number; *SD* = standard deviation; (k)m = (kilo)meter.

Table 2 presents the regression coefficients for the associations between physical functioning and caregiving, controlled for several covariates. Other stepwise associations (Models 1, 2, and 3) are depicted in Supplementary Tables 2–4. According to Table 2, the effect of within-person time on all types of physical functioning and on the total score was significantly negative. This means that, on average, noncaregivers experienced a decline in physical functioning between baseline and W2. The interaction terms of physical functioning and within-person time show that the decline in physical functioning was significantly weakened by caregiving for three outcome variables, namely the overall physical functioning, moderate activities, and lifting and carrying groceries. Specifically, for noncaregivers, overall physical functioning declined over time (*B* = −0.08), and the odds of being unlimited at moderate activities (ExpB = 0.44) and lifting or carrying groceries (ExpB = 0.63) decreased. For caregivers, limitations in overall physical functioning increased to a lesser extent (*B* = 0.01–0.08 = −0.07), and the odds of being unlimited in moderate activities (ExpB = 1.22 * 0.44 = 0.54) and lifting or carrying groceries (ExpB = 0.63 * 1.17 = 0.74) decreased to a lesser extent.

**Table 2:** . Final GEE Model Results for Predicting Change in Physical Functioning Total Score and Each Physical Functioning Type

Independent variables	*B* for total PF score (linear)	Estimated Exp(*B*); ref: limited									
		Vigorous activities	Moderate activities	Lift or carry	Climb > 1 flight stairs	Climb 1 flight stairs	Bend & kneel	Walk > 1 km	Walk 0.5 km	Walk 100 m	Bathe & dress
Wave (ref: baseline)	−0.08^**^	0.64^**^	0.44^**^	0.63^**^	0.54^**^	0.31^**^	0.59^**^	0.48^**^	0.42^**^	0.39^**^	0.57^**^
Caregiving (ref: not being caregiver)	−0.001	0.93	0.96	0.88^*^	0.96	1.12	1.02	.95	1.10	1.16	1.35
Wave 2 × caregiving	.0011^*^	0.99	1.22^*^	1.17^*^	1.12	0.99	1.01	1.13	1.08	0.978	0.89
Gender (ref: man)	−0.03^**^	0.83^**^	0.70^**^	0.44^**^	0.71^**^	0.84^**^	1.03	0.99	1.05^*^	1.12	1.95^**^
Age	−0.01^**^	0.95^**^	0.95^**^	0.87^*^	0.96^**^	0.95^**^	0.97^**^	0.94^**^	0.93^**^	0.94^**^	0.98
High income (ref: low)	0.05^**^	1.15^*^	1.40^**^	1.25^**^	1.30^**^	1.60^**^	1.27^**^	1.60^**^	1.76^**^	1.83^**^	1.49^*^
Medium income (ref: low)	0.02^*^	0.96	1.14^*^	1.08	1.10	1.25^*^	1.07	1.21^*^	1.27^*^	1.31^*^	1.23
High education (ref: low)	0.04^**^	0.99	1.37^**^	1.26^**^	1.27^**^	1.63^**^	1.32^**^	1.44^**^	1.58^**^	1.94^**^	1.65^**^
Medium education (ref: low)	0.02^**^	1.02	1.15^**^	1.14^**^	1.15^**^	1.21^**^	1.15^**^	1.11	1.18^*^	1.24^*^	1.39^*^
Mental impairment present (ref: no)	−0.02^**^	0.95^**^	0.95^**^	0.96^*^	0.85^*^	0.95^**^	0.97^**^	0.89^*^	0.90	0.97	0.93
Physical impairment score	−0.04^**^	0.75^**^	0.75^**^	0.77^**^	0.78^**^	0.80^**^	0.78^**^	0.80^**^	0.80^**^	0.80^**^	0.76^**^

*Note*: PF = physical functioning; ref = reference category.

^*^ *p* < .05.

^**^ *p* < .001.

Regarding the covariates, women participants had poorer overall physical functioning, and more limitations in vigorous and moderate activities, lifting or carrying groceries, and climbing one or more flights of stairs compared to men, whereas man participants had more limitations in walking 0.5 km and in bathing or dressing than women. Higher age had a significant negative association with all physical functioning types and on the total score. Higher household income increased the odds of being unlimited in all physical functioning types and the probability of being more unlimited in the total score. Medium household income had a significant positive association with overall physical functioning, moderate activities, climbing one flight of stairs, and walking various distances. Similarly, medium and high educational attainment also had positive associations with being unlimited in the total score and in all physical functioning types over time, except for vigorous activities and walking more than 1 km. The presence of one or more mental impairments had a negative association with being unlimited in overall physical functioning; vigorous activities; carrying or lifting groceries; climbing stairs; bending, kneeling, or stooping; and walking more than 1 km. Participants with a higher physical impairment score at baseline had significantly more limitations in all physical functioning types and had a lower total score over time.

**Figure 1.**
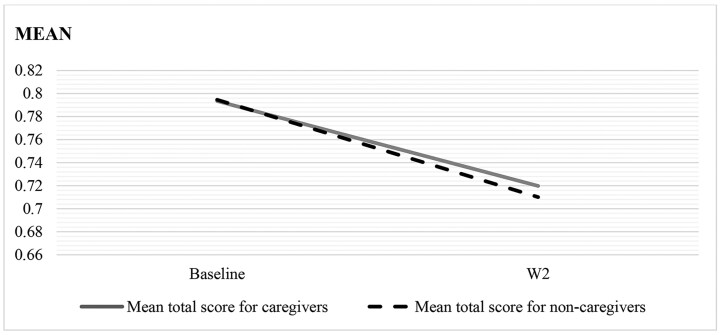
Decline in overall physical functioning for caregivers versus noncaregivers from baseline to W2. The interaction wave × caregiving was significant (*p* < .05) for overall physical functioning.

**Figure 2.**
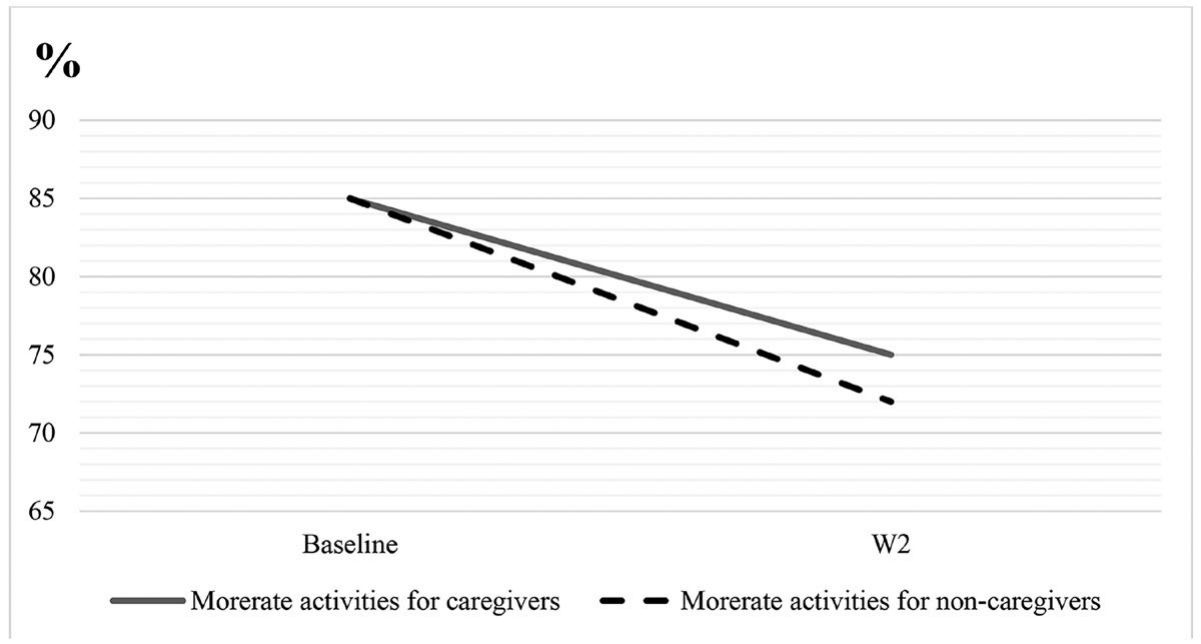
Decline in moderate activities for caregivers versus noncaregivers from baseline to W2. The interaction wave × caregiving was significant (*p* < .05) for moderate activities.

**Figure 3.**
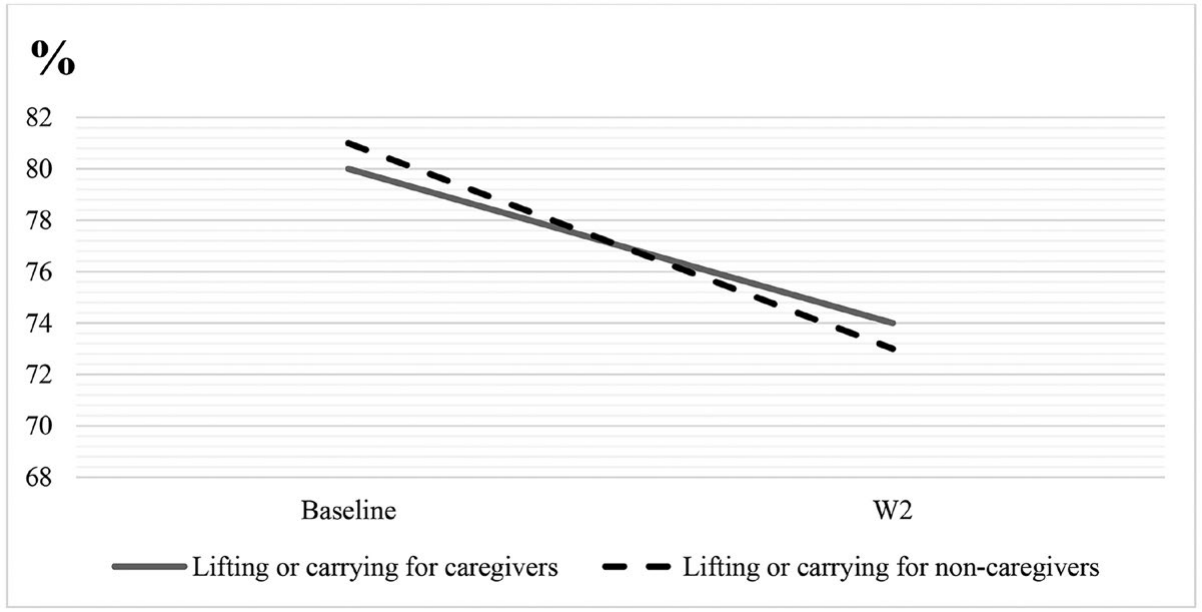
Decline in ability to lift or carry groceries for caregivers versus noncaregivers from baseline to W2. The interaction wave × caregiving was significant (*p* < .05) for lifting or carrying groceries.

The declines in estimated marginal means for overall physical functioning, moderate activities, and lifting or carrying groceries are visualized in Figures 1–3. These figures show that despite noncaregivers having equal or less limitation at baseline, their physical functioning declined more over time compared with caregivers.

## Discussion and Implications

Our study explored the association between informal caregiving in later life and changes in various physical functioning types over time among older adults living in the Northern Netherlands. The results of our study highlight that caregiving lessens the aging-related decline in physical functioning over time. Notably, individuals who were informal caregivers experienced less decline in their overall physical functioning, as well as in their ability to engage in moderate activities and to lift or carry groceries. These results echo previous research suggesting that caregivers tend to maintain better levels of physical functioning over time than those who are not involved in caregiving responsibilities (Fredman et al., 2009; McCann et al., 2004).

The implications of these results shed light on the various declines in each type of physical functioning among caregivers and noncaregivers, particularly concerning moderate activities and lifting and carrying groceries. It is plausible that caregivers engage more frequently in such activities, potentially mitigating physical decline through consistent practice and training. This suggests that the caregiving role itself may serve as a form of physical exercise, contributing toward the maintenance of certain aspects of physical functioning. Housework, which often increases with the caregiving role, involves various physical tasks that we have included as measures of physical functioning in this study. Research suggests that engaging in housework is positively associated with physical and cognitive health in older adulthood (Chu et al., 2023; Lee et al., 2021; Rodriguez-Stanley et al., 2020).

The majority of research indicates a decline in physical functioning among older adults over time (Payette et al., 2010; Young et al., 2002). This trend might relate to the fact that physical decline is inevitable with aging. Our results support this, demonstrating a significant association between older age and decline across all types of physical functioning. The significance of these declines warrants consideration, especially concerning the ability of caregivers to effectively perform their caregiving tasks related to specific physical functioning types, such as vigorous activities, walking, climbing stairs, bathing, and dressing. Thus, addressing the specific physical needs of caregivers becomes paramount to sustaining their health and caregiving responsibilities effectively. It is interesting to note, however, that a few studies suggest a potential improvement in physical functioning among older disabled adults over time (Nikolova et al., 2011; Spalter et al., 2014).

Our findings in relation to mental and physical impairments yielded interesting insights. Poorer mental and physical health is associated with more limitation in physical functioning (Garber et al., 2010; van der Windt et al., 2008). The higher impairment among caregivers versus noncaregivers at baseline was contrary to a part of the healthy caregiver hypothesis, specifically, the part which suggests that healthier people are more likely to start caregiving (Bertrand et al., 2012; Bhattacharyya et al., 2023). However, our findings align with another aspect of the hypothesis, because we observed a smaller decline in physical functioning among caregivers. This suggests that caregivers generally remain healthier than noncaregivers over time. The healthy caregiver hypothesis therefore may not be fully applicable to older caregivers.

It is important to mention that approximately 78% of caregivers at baseline stopped caregiving before W2. This factor might partially explain the lesser decline in physical functioning among caregivers, as studies have shown that ceasing care duties can improve physical health in the long term (Gräsel, 2003). An inference drawn from this result is that caregiving in later life most likely does not extend beyond 10 years. Supporting this, a study on English and Welsh census data between 2001 and 2011 revealed that about two-thirds of caregivers did not continue caregiving after 10 years; those who did continue were mostly women and were aged between 45 and 54 years (Robards et al., 2015).

Our results revealed a significant gender difference among caregivers, with 61% being women. This finding supports investigations conducted by Pinquart and Sörensen (2005, 2006, 2007). Over time, women also exhibited more limitations in overall physical functioning, vigorous and moderate activities, climbing stairs, and lifting or carrying groceries compared with men. In contrast, men became more limited in other physical functioning types, namely walking 0.5 km and bathing or dressing. This finding carries two important implications: First, although women are more likely to become caregivers, and caregiving is linked with maintaining physical functioning, our research indicates that women experience a greater decline in their everyday physical functioning and mobility over time compared with men. This aligns with research by Espelt et al. (2010), who suggested that women may be more susceptible to disabilities in later life than men. Second, our study highlights that examining specific types of physical functioning rather than relying solely on the total score can be more effective in elucidating gender differences in later-life physical health.

Consistent with existing research (see, for instance, Korda et al., 2014; Pinquart & Sörensen, 2005, 2006, 2007; Zheng & George, 2012), we found that higher baseline household income and educational attainment levels were significantly associated with maintaining higher physical functioning (total score and each type) over time. The fact that caregivers were significantly more educated than noncaregivers might provide another explanation for their lesser decline in physical functioning over time. More education and resources help caregivers maintain a healthier lifestyle and better physical functioning in order to weaken the negative health consequences associated with both caregiving and aging, as suggested by Smith et al. (2015) and Sörensen and Conwell (2011).

Our study has some limitations, which should be taken into account in future research. First, our data did not include care status characteristics, such as duration, intensity, level of burden, and the subject of care, which could provide further explanation for our results (Chen et al., 2015; Fredman et al., 2009; Pinquart & Sörensen, 2007). Therefore, our caregiving measure included family care as well as care for neighbors or friends. The type and duration of care, as well as the intensity of physical and mental stress, may differ for these types of caregivers. However, we were unable to distinguish these types in our study. Second, the representativeness of the Lifelines data is limited (Klijs et al., 2015). This limitation prompted us to exclude ethnicity, as over 99% of participants were from one ethnic category, namely White/East and West European. Moreover, the household income and education levels were higher among our final sample when compared with the excluded participants. Third, this longitudinal study was restricted to two data collection waves, which were 10 years apart. Hence, our results are limited in capturing the complexity of temporal patterns in physical functioning over an extended period. Additionally, the relatively long 10-year time interval between data collection waves does not capture the exact duration of caregiving and the reasons for cessation. We recommend further research on caregiving duration, as it is essential for including caregivers in economic evaluations and health policy (Urwin et al., 2021). Subjective reports of physical functioning and other variables provide valuable insights in our study. However, relying solely on subjective data introduces limitations, such as potential biases and the inability to verify accuracy objectively. Integrating objective measures alongside subjective reports can enhance the completeness of results in future research. Lastly, despite including only baseline independent variables in the regression model, reverse causality remains a concern due to the inherent characteristics of cohort data.

Notwithstanding these limitations, this study makes meaningful contributions to both research and practice. Our results provide insight into previously unexplored aspects that deepen our understanding of healthy aging, informal care, and disability in later life. Specifically, this study represents one of the initial investigations into diverse types of physical functioning, aiming to assess the mobility of a specific, often vulnerable, and growing population group, namely older informal caregivers. Unlike previous approaches that rely solely on total scores, our examination provides a more nuanced understanding of this group’s ability to be mobile. Moreover, our longitudinal method addresses certain gaps in existing studies. Specifically, it accounts for the change in physical functioning over time and it mitigates the risk of reverse causality when examining associations. Focusing on self-reported physical functioning in our study complements the physical performance measurements. Apart from clinical researchers, our approach in distinguishing between various types of physical functioning could also be used by orthopedic specialists and physiotherapists to gain a specified perspective on the decline or improvement of mobility among older caregivers. Additionally, our approach underscores the importance of considering caregivers’ demographic characteristics and mental and physical health at baseline when assessing changes in physical functioning. Overall, this research contributes valuable insights into healthy aging, informal care, and disability in later life, recommending the need for tailored interventions and policies for a growing population of older adult caregivers.

## Supplementary Material

gnaf108_suppl_Supplementary_Materials

## Data Availability

The data used in this research is not publicly available but we will be happy to share details about our analysis methods and syntaxes. This study was not pre-registered.
