# Supplementary material for: Associations Between Informal Caregiving and Physical Functioning: A Longitudinal Analysis of Dutch Older Adults
Source: Gerontologist. 2025 Mar 18;65(6):gnaf108. doi: 10.1093/geront/gnaf108 (PMC12138341; doi:10.1093/geront/gnaf108)
Supplement: gnaf108_suppl_Supplementary_Materials [file gnaf108_suppl_supplementary_materials.docx]

**SUPPLEMENTARY MATERIAL**

**Supplementary Table 1**. Status of physical functioning types at baseline and W2

|  | Baseline frequency (%) | | W2 frequency (%) | |
| --- | --- | --- | --- | --- |
| Physical functioning aspects | Limited | Unlimited | Limited | Unlimited |
| Vigorous activities, such as running, lifting heavy objects, participating in strenuous sports | 66.4 | 33.6 | 75.0 | 25.0 |
| Moderate activities, such as moving a table, pushing a vacuum cleaner, cycling | 16.4 | 83.6 | 27.4 | 72.6 |
| Lifting or carrying groceries | 21.3 | 78.7 | 27.7 | 72.3 |
| Climbing several flights of stairs | 24.1 | 75.9 | 34.8 | 65.2 |
| Climbing one flight of stairs | 7.8 | 92.2 | 19.8 | 80.2 |
| Bending, kneeling, or stooping | 32.9 | 67.1 | 44.2 | 55.8 |
| Walking more than 1 km | 12.3 | 87.7 | 21.1 | 78.9 |
| Walking 0.5 km | 5.9 | 94.1 | 12.0 | 88.0 |
| Walking 100 m | 3.1 | 96.9 | 7.5 | 92.5 |
| Bathing or dressing yourself | 1.9 | 98.1 | 3.4 | 96.6 |

| **Supplementary Table 2**. GEE Model 1 regression coefficients | Estimated Exp(B); ref: limited | Bathe & dress | .58** | 1.44* | .89 | ** P<0.05; ** P<0.001*  *Note.* PF = physical functioning; ref = reference category  ***p* < .001. **p* < .05 & *p* > .001. |
| --- | --- | --- | --- | --- | --- | --- |
|  |  | Walk 100 m | .40** | 1.18 | .97 |  |
|  |  | Walk 0.5 km | .45** | 1.12 | 1.06 |  |
|  |  | Walk >1 km | .51** | .95 | 1.12 |  |
|  |  | Bend & kneel | .62** | .99 | 1.01 |  |
|  |  | Climb 1 flight stairs | .35** | 1.08 | .99 |  |
|  |  | Climb >1 flight stairs | .57** | .90* | 1.10 |  |
|  |  | Lift or carry | .67** | .79** | 1.14* |  |
|  |  | Moderate activities | .49** | .90 | 1.19* |  |
|  |  | Vigorous activities | .66** | .88* | .99 |  |
|  | B for total PF score (linear) | | -.08** | -.01 | .01* |  |
|  |  | Independent variables | Wave (ref: baseline) | Caregiving (ref: not being caregiver) | Wave 2 × caregiving |  |

| **Supplementary Table 3**. GEE Model 2 regression coefficients | Estimated Exp(B); ref: limited | Bathe & dress | .58** | 1.38* | .89 | 1.26* | .98 | ** P<0.05; ** P<0.001*  *Note.* PF = physical functioning; ref = reference category  ***p* < .001. **p* < .05 & *p* > .001. |
| --- | --- | --- | --- | --- | --- | --- | --- | --- |
|  |  | Walk 100 m | .40** | 1.18 | .97 | .74** | .94** |  |
|  |  | Walk 0.5 km | .44** | 1.11 | 1.07 | .72** | .93** |  |
|  |  | Walk >1 km | .50** | .96 | 1.12 | .71** | .94** |  |
|  |  | Bend & kneel | .62** | 1.02 | 1.01 | .76** | .98** |  |
|  |  | Climb 1 flight stairs | .34** | 1.13 | .99 | 61.** | .95** |  |
|  |  | Climb >1 flight stairs | .57** | .97 | 1.11 | .51** | .96** |  |
|  |  | Lift or carry | .66** | .89* | 1.15* | .34** | .97** |  |
|  |  | Moderate activities | .48** | .97 | 1.20* | .51** | .95** |  |
|  |  | Vigorous activities | .66** | .91 | .99 | .64** | .96** |  |
|  | B for total PF score (linear) | | -.08** | -.001 | .01* | -.07** | -.001** |  |
|  |  | Independent variables | Wave (ref: baseline) | Caregiving (ref: not being caregiver) | Wave 2 × caregiving | Gender (ref: man) | Age |  |

| **Supplementary Table 4**. GEE Model 3 regression coefficients | Estimated Exp(B); ref: limited | Bathe & dress | .58** | 1.33 | .89 | 1.50** | .99 | 1.89** | 1.44* | 1.87** | 1.44* | ** P<0.05; ** P<0.001*  *Note.* PF = physical functioning; ref = reference category  ***p* < .001. **p* < .05 & *p* > .001. |
| --- | --- | --- | --- | --- | --- | --- | --- | --- | --- | --- | --- | --- |
|  |  | Walk 100 m | .40** | 1.14 | .97 | .89 | .94** | 2.13** | 1.46** | 2.11** | 1.28* |  |
|  |  | Walk 0.5 km | .44** | 1.09 | 1.07 | .83 | .93** | 2.03** | 1.41** | 1.70** | 1.21* |  |
|  |  | Walk >1 km | .49** | .95 | 1.12 | .8** | .94** | 1.81** | 1.32** | 1.54** | 1.13* |  |
|  |  | Bend & kneel | .61** | 1.00 | 1.01 | .82** | .98** | 1.45** | 1.18* | 1.39** | 1.17** |  |
|  |  | Climb 1 flight stairs | .34** | 1.10 | .99 | .68** | .95** | 1.80** | 1.37** | 1.74** | 1.23** |  |
|  |  | Climb >1 flight stairs | .56** | .96 | 1.11 | .57** | .97** | 1.49** | 1.21* | 1.35** | 1.16** |  |
|  |  | Lift & carry | .66** | .88* | 1.15* | .37** | .97** | 1.44** | 1.20* | 1.36** | 1.16** |  |
|  |  | Moderate activities | .47** | .95 | 1.20* | .55** | .96** | 1.56** | 1.26** | 1.48** | 1.17** |  |
|  |  | Vigorous activities | .65** | .91* | .99 | .67** | .96** | 1.32** | 1.07 | 1.08 | 1.04 |  |
|  | B for total PF score (linear) | | -.08** | -.003 | .01* | -.06** | -.01** | .07** | .04** | .05** | .02** |  |
|  |  | Independent variables | Wave (ref: baseline) | Caregiving (ref: not being caregiver) | Wave 2 × caregiving | Gender (ref: man) | Age | High income (ref: low) | Medium income (ref: low) | High education (ref: low) | Medium education (ref: low) |  |
